# Wnt-signalling pathway in ovarian epithelial tumours: increased expression of *β*-catenin and GSK3*β*

**DOI:** 10.1038/sj.bjc.6601265

**Published:** 2003-09-30

**Authors:** K Rask, A Nilsson, M Brännström, P Carlsson, P Hellberg, P-O Janson, L Hedin, K Sundfeldt

**Affiliations:** 1 Department of Physiology, Göteborg University, Göteborg, Sweden; 2 Department of Obstetrics and Gynaecology, Göteborg University, Göteborg, Sweden; 3 Department of Molecular Biology, Göteborg University, Göteborg, Sweden; 4 Department of Anatomy and Cell Biology, University of Bergen, Bergen, Norway

**Keywords:** Wnt-signalling, normal ovary, ovarian cancer, *β*-catenin, GSK3*β*, Tcf/Lef, APC

## Abstract

Beta-catenin is involved in both cell–cell adhesion and in transcriptional regulation by the Wingless/Wnt signalling pathway. Alterations of components of this pathway have been suggested to play a central role in tumorigenesis. The present study investigated, by immunohistochemistry and immunoblotting, the protein expression and localisation of *β*-catenin, adenomatous polyposis coli (APC), glycogen synthase kinase 3*β* (GSK3*β*) and lymphocyte enhancer factor-1 (Lef-1) in normal human ovaries and in epithelial ovarian tumours *in vivo* and *in vitro*. Immortalised human ovarian surface epithelium and ovarian cancer cell cells (OVCAR-3) expressed *β*-catenin, APC, GSK3*β* and Lef-1. Nuclear staining of *β*-catenin and Lef-1 were demonstrated only in OVCAR-3 cells. There were significant increases of *β*-catenin and GSK3*β*, while APC was reduced in ovarian cancer compared to the normal ovary. Beta-catenin and Lef-1 were coimmunoprecipitated in ovarian tumours, but not in the normal ovary. Nuclear localisation of *β*-catenin or Lef-1 could not be demonstrated in the normal ovary or in the ovarian tumours. The absence of nuclear localisation of *β*-catenin could be due to an increased binding to the cadherin–*α*-catenin cell adhesion complex. In fact, we have earlier reported an increased expression of E-cadherin in ovarian adenocarcinomas. In summary, this study demonstrates an increase in the expression of components of the Wingless/Wnt pathway in malignant ovarian tumours. The increase suggests a role for this signalling pathway in cell transformation and in tumour progression. However, it remains to be demonstrated whether it is an increased participation of *β*-catenin in transcriptional regulation, or in the stabilisation of cellular integrity, or both, that is the crucial event in ovarian tumorigenesis.

The cytoplasmic protein *β*-catenin, initially discovered as a component of cell–cell adhesive junctions, has also been demonstrated to be a critical downstream mediator of Wnt-signalling (Giarré *et al*, 1998). Beta-catenin is phosphorylated by glycogen synthase kinase 3*β* (GSK3*β*), in connection with the tumour-suppressor gene product adenomatous polyposis coli (APC) and Axin. The formation of this complex depends on the GSK3*β* phosphorylation status. In the normal cell, degradation of *β*-catenin through the ubiquitin pathway is facilitated by phosphorylation (BenZeev and Geiger, 1998). Mutational changes to the complex will lead to an increased free pool of *β*-catenin in the cytoplasm. Apart from being important for cell–cell adhesion, *β*-catenin also binds to the transcription factor T-cell factor/lymphocyte enhancer factor (Tcf/Lef) either in the cytoplasm or in the nucleus. This is followed by transcriptional activation of target genes, that is, c-*myc*, E-cadherin and cyclin D1 (reviewed by Roose and Clevers, 1999). In adherens junctions, *β*-catenin binds directly to the cytoplasmic tail of cadherins and will thereby stabilise cell–cell adhesion. The regulation of this dual function of *β*-catenin is not clear (Giarré *et al*, 1998).

The majority of ovarian tumours are epithelial-derived adenomas or adenocarcinomas. They are believed to arise from inclusion cysts situated in the ovarian stroma and will typically grow in cystic formations or solid formations (Scully, 1995). The adenocarcinomas invade the nearby pelvic surroundings and later the abdomen by direct growth or via ascites fluid. Haematogenous spread with peripheral metastases, for example, to the liver and lung, are rare.

Cell–cell adhesion has been implicated to be of great importance for the invasive capacity of most tumour types. The downregulation of cadherins or dysfunction of the cadherin–catenin complex has been noted in different types of tumours. In the human ovary, neuronal (N) cadherin, but not epithelial (E) cadherin, is expressed in the normal ovarian surface epithelium (OSE) (Sundfeldt *et al*, 1997; Auersperg *et al*, 2001). However, E-cadherin is upregulated in inclusion cysts of the normal ovary and in ovarian tumours with invasive capacity (Sundfeldt *et al*, 1997; Auersperg *et al*, 2001). At the protein level, both *α*- and *β*-catenin are expressed in normal OSE and invasive ovarian tumours (Auersperg *et al*, 2001). Mutations of the *β*-catenin gene at the binding site for GSK3*β* was demonstrated in 16–30% of the endometrioid type of ovarian adenocarcinomas, but not in other types of ovarian tumours (Gamallo *et al*, 1999; Wright *et al*, 1999). GSK3*α* and GSK3*β* mRNAs have been described in normal human ovaries (Lau *et al*, 1999). The expression of GSK3 has, to our knowledge, not been investigated in ovarian tumours previously. Mutations of APC, considered to be a tumour suppressor, predispose for both sporadic and inherited colon cancers. It is not known whether APC is mutated in ovarian tumours or not. However, the expression of APC has been demonstrated in normal OSE (Midgley *et al*, 1997), with less staining in 72 out of 113 serous ovarian carcinoma (Karbova *et al*, 2002) and in a large variety of other tumours (Smith *et al*, 1993).

The aim of this study was to analyse components of the Wnt-signalling pathway in normal human ovaries and in ovarian tumours of epithelial origin. Cell-specific protein expression and protein–protein interactions were investigated in normal ovaries, benign and malignant tumours.

## MATERIALS AND METHODS

### Human tissue

Biopsies from normal ovaries and epithelial ovarian tumours were obtained from patients undergoing laparotomy (approved by the Ethics Committee, Göteborg University, Sweden). Tissues were immediately washed in ice-cold 0.9% NaCl, snap frozen in liquid nitrogen and stored at −70°C until analysis. Ovarian tumours were classified according to surgical staging. All samples were examined by two independent and experienced gynaecological pathologists for histological diagnosis and grade (Table 1

**Table 1:** Histological description

		**Stage**		
**Type/grade**	***n***	**I**	**II**	**III**
*Normal*	*10*			
Fertile	4			
Postmenopausal	6			
				
*Thecom*	*2*			
				
*Benign adenoma*	*6*			
Serous	4			
Mucinous	2			
				
*Borderline tumour*	*5*			
Serous	4	4		
Mucinous	1	1		
				
*Adenocarcinoma*	*24*			
Serous	23			
High diff	2	1		1
High/moderately diff	2			2
Moderately diff	6	1		5
Moderately/poor diff	6		2	4^a^
Poor diff	5	1		4^b^
Undiff	2			2
Mucinous	1	1		
				
Total	47	9	2	18

Italics represent totals for each category. diff=differenciated.

a +3 metastases investigated.

b +1 metastasis investigated.

). An SV-40 immortalised ovarian surface epithelial (IOSE) cell line (a gift from Professor Auersperg, University of British Columbia, Canada) and the cancer cell line, NIH-OVCAR-3 (no. HTB161, ATCC, Rockville, MD, USA) were used. One colorectal cancer (CRC), Dukes stage A and one normal colorectal biopsy from the same patient was used as control for the anti-APC antibody.

### Immunoblotting

Soluble tissue and cells were prepared by homogenisation in a PE-buffer with proteinase inhibitors, analysed for protein content by the method of Lowry and stored at −70°C as previously described (Sundfeldt *et al*, 1999). Briefly, 35 *μ*g of total protein was diluted in a sodium dodecyl suplhate (SDS)-sample buffer and heated before loading on a 1D SDS-polyacrylamide gel (8% Tris-Glycine) or NuPage gel (10%) (NOVEX, San Diego, CA, USA). Proteins were transferred onto a polyvinyldifluoride (PVDF) membrane (Amersham, Buckinghamshire, UK) using a blotting system (NOVEX). The membrane was then incubated with antibodies against *β*-catenin (monoclonal, 1 : 1000), GSK3*β*, (monoclonal, 1 : 500) (Transduction Laboratories, Nottingham, UK), *α*-catenin (monoclonal, 1 : 1000; Alexis, Lausen, Switzerland) APC-COOH terminus or APC-NH_2_-terminus (polyclonal, 1 : 500; Santa Cruz Biotechnology, Santa Cruz, CA, USA), Tcf-Lef (polyclonal) and Lef-1 (monoclonal, 1 : 500; Kamiya Biomedical Company, Seattle, WA, USA) or Lef-1 antiserum (1 : 10000) (Carlsson *et al*, 1993). Prestained standards (SeeBlue; NOVEX) were used as weight markers. Immunoreactive proteins were visualised by chemiluminescence using alkaline phosphatase-conjugated secondary antibodies (Santa Cruz Biotechnology) and CDP-star (Tropix, Bedford, MA, USA) as substrate.

### Densitometric scanning

Semiquantitative measurements of proteins from the immunoblots were made by densitometry (Fluor-S™ Multimager, Quantity One ver. 4.1.0., BioRad, Hercules, CA, USA). The optical density (OD) of each band was measured. For quantification, a reference sample, same on each blot, was included. The standard was set to 100%. The signal from each band was then correlated to the standard and this relative number was used in the statistical analysis.

### Immunohistochemistry (IHC)

Fresh frozen tissues were cryosectioned and fixed in cold acetone for 10 min, air dried and stored at −20°C as previously described (Sundfeldt *et al*, 1999). Briefly, slides were blocked with 5% nonfat milk (NFM) before the addition of primary antibodies to *β*-catenin (1 : 100), Lef-1 (1 : 100), Lef-1 antiserum (1 : 100), GSK3*β* (1 : 50), APC-COOH and APC-NH_2_-terminus (1 : 100) and incubated overnight. Bound antibodies were visualised by biotinylated secondary horse anti-mouse or goat anti-rabbit antibodies (1 : 200; Vector, Burlingham, CA, USA) and Streptavidin-FITC (1 : 200; Amersham, Buckinghamshire, UK). In control sections, which showed negligible signals, the first antibody was replaced by normal mouse IgG (Jackson Immuno Research, West Grove, PN, USA) or 5% NFM (Figure 4G and I). Sections were photographed with a Nikon microphot FX fluorescence microscope. To verify the epithelial origin of cells, all samples were stained with anti-keratin AE1/AE3 monoclonal antibody (1 : 40) (cat. no. 1124161, Boehringer Mannheim, Germany) (Figure 4H).

### Immunoprecipitation

Immunoprecipitations were performed with the Seize-It Mammalian Primary Antibody Precipitation kit (Pierce, Rockford, IL, USA) and Catch-and-Release (Upstate Biotechnology, Charlottesville, VA, USA) according to the protocols provided by each manufacturer, with modifications. Briefly, a soluble lysate of an ovarian serous adenocarcinoma, poorly differentiated, stage III, was prepared as described above with the exception of the homogenisation buffers that were provided with the kits. The lysates (1 mg of total protein per sample) were incubated with the primary antibody overnight at 4°C on a rotating platform. The eluted fractions were concentrated by ethanol precipitation (Current Protocols, Wiley & Sons) and vacuum-dried. The pellets were dissolved in an SDS-sample buffer. Separation by SDS–PAGE and immunodetection was performed as described above.

### Statistics

The nonparametric Kruskal–Wallis test for comparing groups was followed by the *post hoc* Wilcoxon rank-sum test and used in the analysis of immunoblotting data obtained by the densitometric scanning of membranes. A *P*-value less than 0.05 was considered to be significant. Values are given as mean±s.e.m.

## RESULTS

### Intracellular localisation of *β*-catenin, Lef-1, GSK3*β* and APC in IOSE and OVCAR-3

**Figure 1:**
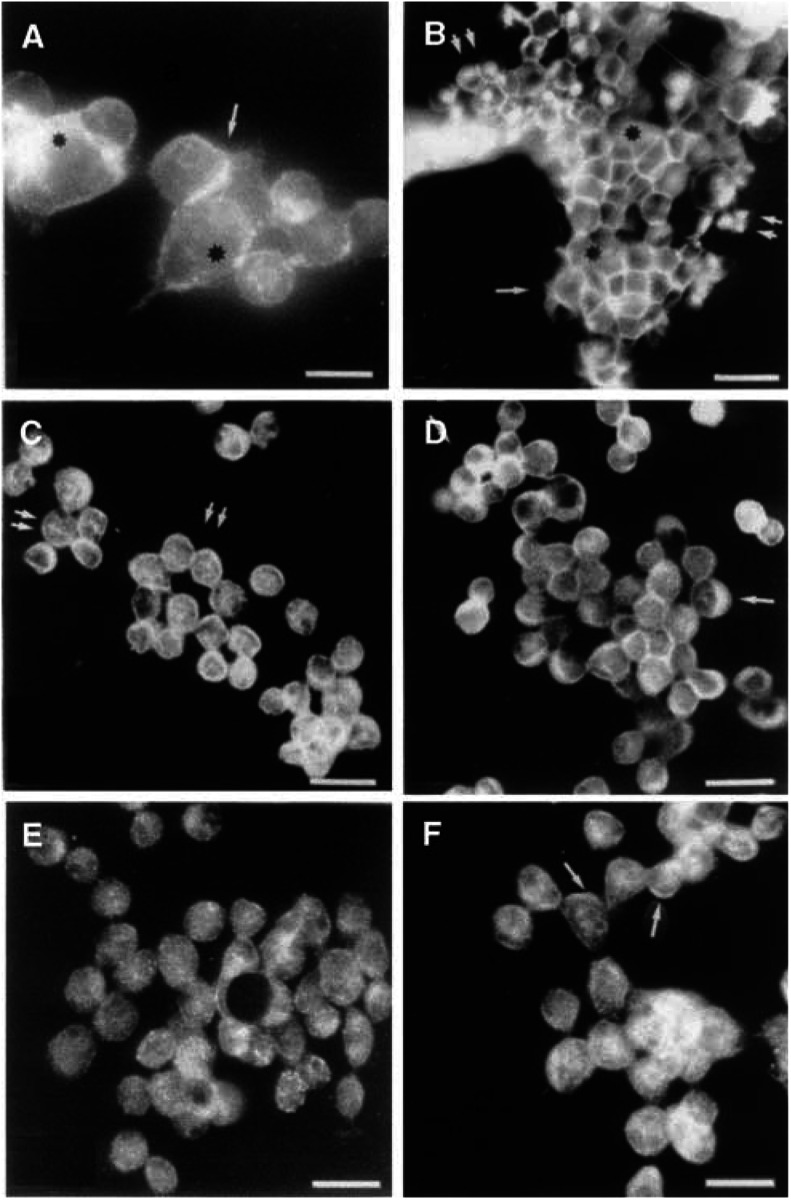
Immunohistochemistry analysis of (**A**) normal IOSE, (**B–E**) ovarian cancer cell line OVCAR-3, stained with antibodies directed towards; (**A, B**) *β*-catenin, (**C**) Lef-1, (**D**) GSK3*β*, (**E**) APC-COOH terminus and (**F**) APC-NH_2_ terminus. See text for explanation of arrows and stars. Bar=50 *μ*m.

The normal IOSE cells demonstrated staining of *β*-catenin at the cell borders (single arrow and black stars (Figure 1A

). In OVCAR-3, *β*-catenin was localised to the nucleus of some cell clusters (double arrows, Figure 1B) in addition to the general staining of the cell membranes (single arrow and black star, Figure 1B). Lef-1 was present in the nucleus with a punctuated staining pattern (double arrows; Figure 1C). However, cytoplasmic staining was more prominent for Lef-1, APC and GSK3*β* in OVCAR-3 cells (Figure 1D, arrows; not indicated in E). GSK3*β* and APC was concentrated to the cell borders of some OVCAR-3 cells (Figure 1D, black star and Figure 1F, arrows). Two antibodies against APC were used (APC-COOH and APC-NH_2_) in the analyses and anti-APC NH_2_-terminus was found to be the most reliable for both IHC and immunoblots. This antibody recognizes full-length APC and truncated APC (Reinacher-Schick and Gumbiner, 2001).

### Increased expression of *β*-catenin and GSK3*β* in ovarian adenocarcinomas

**Figure 2:**
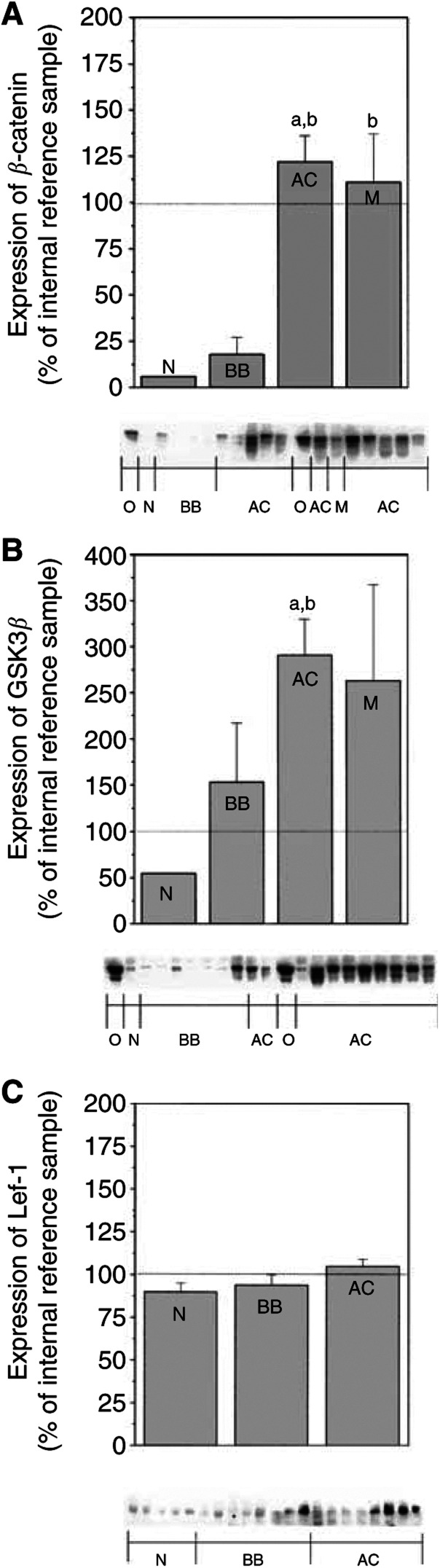
Beta-catenin, GSK3*β* and Lef-1 protein expression presented with histograms and representative immunoblots. The semiquantitative measurements are expressed as mean±s.e.m. and as percentage changes from an internal reference sample, the same for each gel. Kruskal–Wallis, followed by the Wilcoxon rank-sum test as *post hoc* test, was used for statistical analysis of the data obtained by densitometric scanning. a – *P*<0.05 *vs* normal (N) b – *P*<0.05 *vs* benign/borderline (BB). (**A, B**) O=OVCAR-3 cells, N=normal ovary (*n*=2), BB=benign serous adenoma/adenofibroma and borderline type tumour (*n*=8), AC=adenocarcinoma; highly, moderately and poorly differentiated (*n*=21), M=peritoneal metastasis (*n*=4). An undifferentiated carcinoma (not included in the histogram) was used as an internal reference sample. (**A**) Expression of *β*-catenin. (**B**) Expression of GSK3*β*. (**C**) Expression of Lef-1. N=normal ovary (*n*=7), BB=benign serous adenoma/adenofibroma and borderline-type tumour (*n*=9), AC=adenocarcinoma; highly, moderately and poorly differentiated (*n*=20). One of the adenocarcinomas was used as an internal reference sample.

The expression of *β*-catenin and GSK3*β* was analysed by immunoblotting in normal ovaries, benign adenomas, borderline tumours and adenocarcinomas. Statistical analysis revealed significant increases of *β*-catenin (*P*<0.01) and GSK3*β* (*P*<0.001) in the group of ovarian adenocarcinomas (AC) as compared to the group of normal ovaries (N) and benign adenomas/borderline tumours (BB) (Figure 2A and B

).

Lef-1 was expressed in all samples analysed. Even though higher levels of Lef-1 were demonstrated in malignant ovarian tumours, this increase was not significant compared to normal and benign ovarian tumours (Figure 2C). The estimated molecular weights of the proteins expressed in these tumours were as expected: 92 kDa for *β*-catenin, 46–48 kDa for GSK3*β* and approximately 55–60 kDa for Lef-1.

### Expression of APC in normal ovaries and ovarian tumours

**Figure 3:**
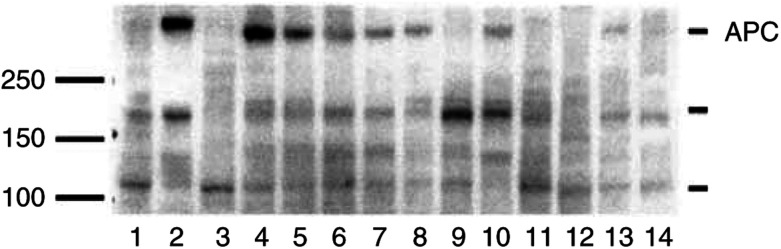
Adenomatous polyposis coli protein expression in ovarian tissue samples presented with a representative immunoblot: (1) colon tumour, Duke A, (2) normal colon mucosa from the patient with a Duke A tumour, (3) OVCAR-3 cells, (4–6) normal ovary, (7, 8) benign adenoma, (9, 10) borderline-type tumour, (11–14) serous adenocarcinoma. The standard marker is indicated at 100–150–250 kDa. Adenomatous polyposis coli indicated at approximately 312 kDa.

APC was expressed in seven out of 12 ovarian samples examined. As a control of APC expression, a normal colon sample and a colorectal carcinoma (CRC; Duke A) from the same patient was included. The normal colon sample, normal ovaries, benign adenomas and borderline-type tumours exhibited higher expression of a band at the expected molecular weight (312 kDa) compared to the adenocarcinomas, OVCAR-3 cells and CRC (Figure 3

). Three-fourths of the ovarian carcinomas, half of the borderline tumours, OVCAR-3 cells and the CRC had none or very low expression at 312 kDa. Bands of unknown importance that could represent truncated APC gene products were also found at approximately 180 and 110 kDa.

### Cell-specific localisation of *β*-catenin and Lef-1 in the normal ovary and ovarian tumours

**Figure 4:**
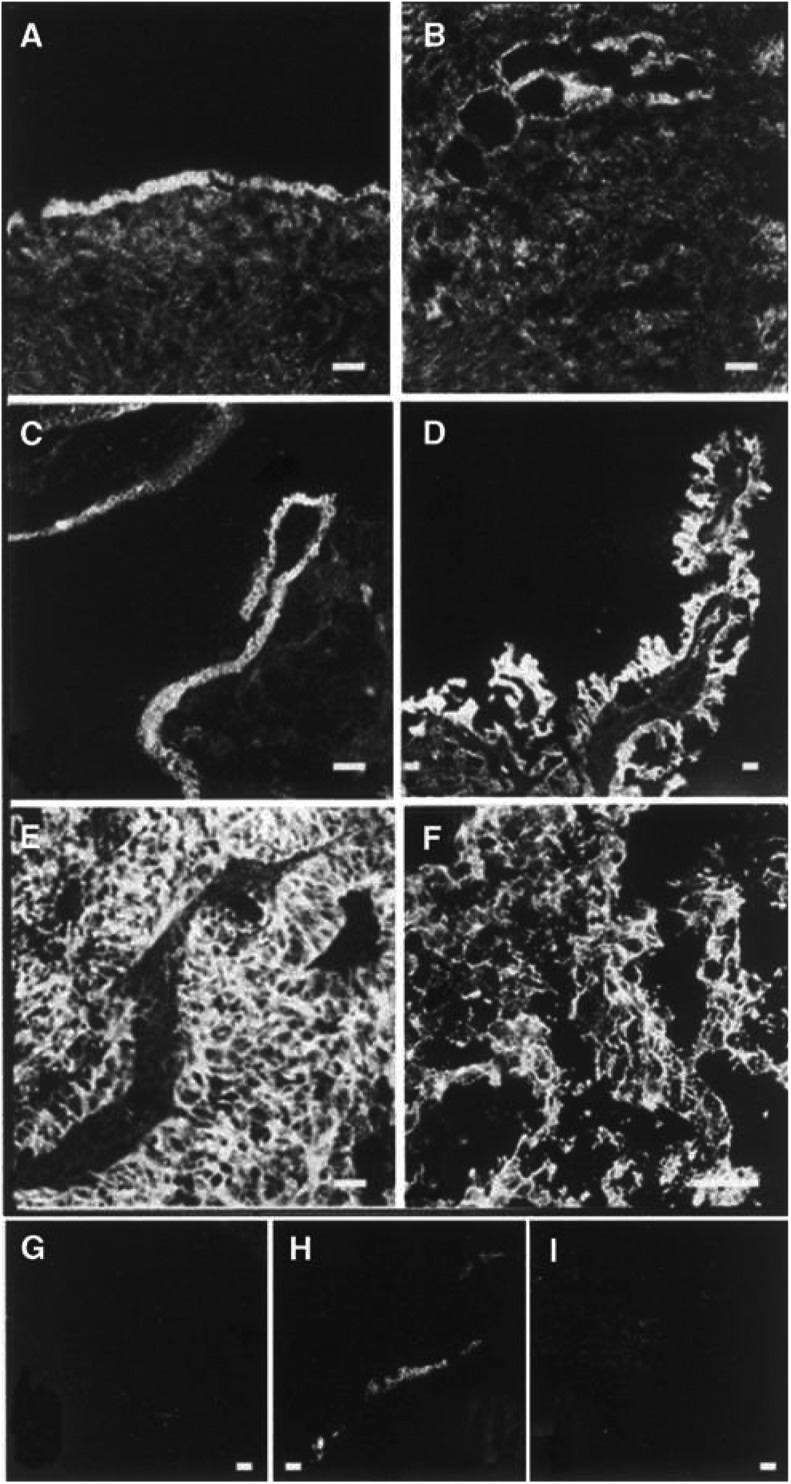
Staining with anti-*β*-catenin in normal human ovaries and in ovarian tumours. Immunohistochemical analysis of (**A**) normal ovary with a stained surface epithelium, (**B**) inclusion cysts located in the normal ovarian stroma lined with epithelial cells, (**C**) borderline-type tumour, (**D**) peritoneal metastasis from a moderately/poorly differentiated adenocarcinoma, (**E**) adenocarcinoma moderately/poorly differentiated; stage III, (**F**) undifferentiated carcinoma, stage III. (**G, H**) Sections of A: (**G**) negative control with IgG as primary antibody, (**H**) positive control with cytokeratin as the primary antibody. (**I**) Section of F: negative control with 5% nonfat milk as the primary antibody. Bar=50 *μ*m.

Normal ovaries from eight patients were stained for *β*-catenin. In OSE and in epithelial cells lining inclusion cysts, *β*-catenin was localised at the cell borders (Figure 4A and B

). Two benign adenomas, four borderline tumours and 18 adenocarcinomas (serous, mucinous and undifferentiated) of different stages and grades exhibited strong and specific *β*-catenin staining in the epithelial cells of the tumours (Figure 4C–F). Staining for *β*-catenin was mainly confined to the cell borders in benign adenomas, borderline and high grade tumours. In one metastasis from a moderately differentiated serous adenocarcinoma and one undifferentiated carcinoma, more diffuse or punctuated staining of the cytoplasmic compartment was present (Figure 4D and F). There was, in contrast to the OVCAR-3 cells, no staining of the nucleus in the tumour cells.

**Figure 5:**
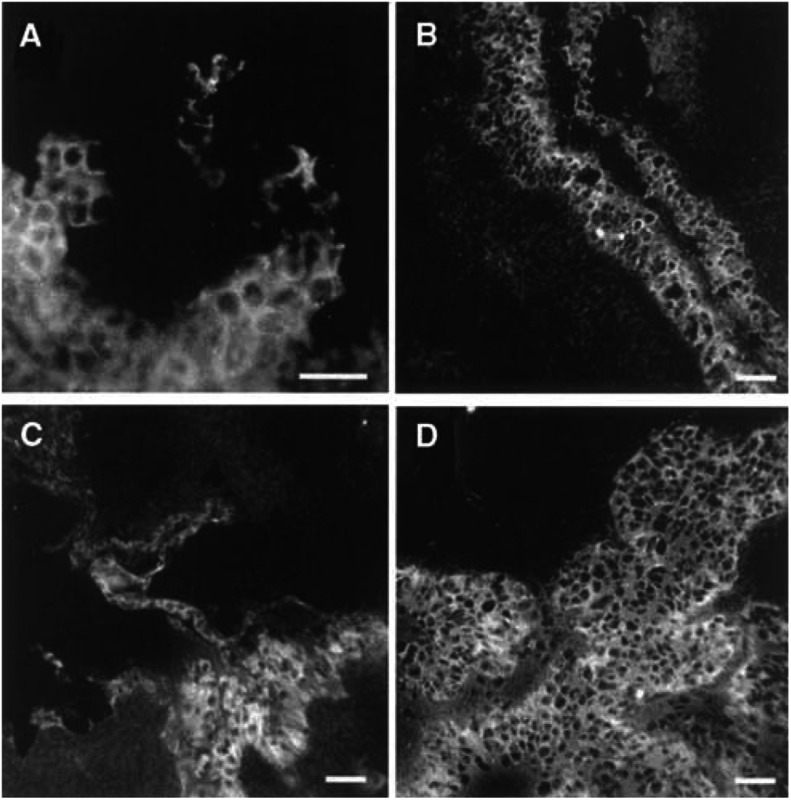
Staining with anti-Lef-1 in a normal human ovary and in ovarian tumours. Immunohistochemical analysis of (**A**) normal ovary with surface epithelium, (**B**) benign serous cyst adenoma, (**C**) borderline-type tumour, and (**D**) serous adenocarcinoma, poorly differentiated. Bar=50 *μ*m.

Lef-1 was localised to the OSE cells in two normal ovaries. Staining of Lef-1 was confined to the cytoplasm and cell borders of these cells and in the epithelial-derived tumour cells of two benign adenomas, two borderline tumours. Lef-1 dominated in the cytoplasm of the more malignant tumour cells including three serous ovarian adenocarcinomas (stage III) and one undifferentiated adenocarcinoma (stage III) (Figure 5A–D

). The dotted staining of the nucleus found in OVCAR-3 cells could not be seen in the tumour biopsies.

### Protein–protein interaction between *β*-catenin and Lef-1

**Figure 6:**
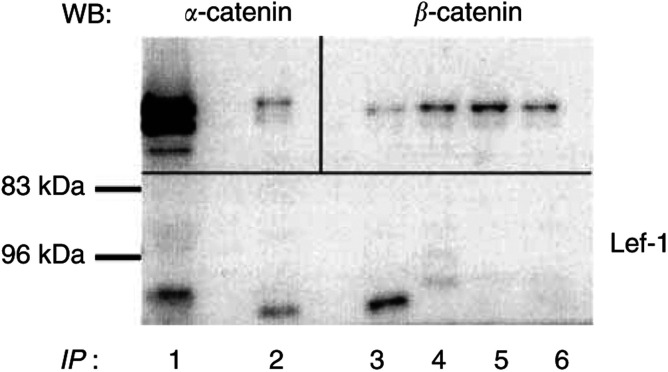
Immunoprecipitation (IP) of extracts from a serous adenocarcinoma (poorly differentiated, stage III). Lane (1) tissue extract, not subjected to IP. Lanes 2–6, IP with the following antibodies; (2) *β*-catenin, (3) Lef-1, (4) Tcf/Lef, (5) *α*-catenin, (6) axin. The precipitates were immunoblotted with *α*-catenin (upper left section), *β*-catenin (upper right section), and Lef-1 (lower section).

Immunoprecipitation was performed in order to detect a physical interaction between *β*-catenin, *α*-catenin and Lef-1 (Figure 6

). A serous adenocarcinoma was immunoprecipitated with *β*-catenin, Lef-1, Tcf/Lef, *α*-catenin or Axin and blotted for the detection of *α*-catenin, *β*-catenin or Lef-1 (Figure 6). The same sample was also subjected to immunoblotting without immunoprecipitation (Figure 6, lane 1) to serve as a control for immunodetection. The immunoprecipitate with *β*-catenin contained one band for Lef-1 with the expected molecular weight of approximately 60 kDa (Figure 6, lane 2). This band was also present in the homogenate not subjected to immunoprecipitation (Figure 6, lane 1). In line with this, *β*-catenin/Lef-1 was co-precipitated in OVCAR-3 cells, but not in a normal ovary (data not shown).

In the *β*-catenin immunoprecipitates, expression of GSK3*β* or APC was not detected (data not shown). However, silver staining of the gel for the immunoprecipitates after transfer revealed a band with a molecular weight of approximately 300 kDa in the adenocarcinoma, suggesting APC as an interacting partner with *β*-catenin in this tumour, in addition to Lef-1 (data not shown). In addition, *β*-catenin was also immunoprecipitated with *α*-catenin (*M*_r_=102 kDa; Figure 6, lane 2). The reverse was also demonstrated (Figure 6, lane 5). This emphasises the dual role for *β*-catenin in these cells.

The immunoprecipitation with Lef-1 in the ovarian tumour sample also contained a band for *β*-catenin at the expected size of 92 kDa (lane 3). The *β*-catenin band was also present in immunoprecipitates with Tcf/Lef and Axin (lanes 4 and 6).

## DISCUSSION

In recent years, it has become clear that *β*-catenin, earlier only known as an important protein in cadherin-mediated cell–cell adhesion, also plays a crucial role in Wnt-signalling. In the normal cell, binding of *β*-catenin to APC, GSK3*β* and Axin will lead to degradation of *β*-catenin through the ubiquitin–proteasome pathway. The importance of *β*-catenin in the Wnt-pathway was originally described in colon and melanoma cells (Morin *et al*, 1997; Rubinfeld *et al*, 1997). We hypothesised that the normal OSE cells also express proteins in the Wnt-signalling pathway and that their deregulation could be of importance for ovarian tumorigenesis.

Ovarian surface epithelium is commonly believed to be the origin for ovarian adenocarcinomas, which arise in inclusion cysts lined with OSE (Scully, 1995). In our study, normal OSE lining the surface of the ovary and the inclusion cysts expressed *β*-catenin, GSK3*β*, APC and Lef-1. Their localisation to these specific epithelial cells was demonstrated by immunohistochemistry. Others, as well as the results of this study, have revealed that *β*-catenin is localised at the cell borders and not in the nucleus of normal OSE or OSE lining the inclusion cysts (Davies *et al*, 1998). However, nuclear *β*-catenin has been described in normal cells of other organs such as the endometrium of the uterus (Nei *et al*, 1999). Mutations in the *β*-catenin gene are infrequent in ovarian carcinoma and interestingly only described in the endometrioid type of epithelial ovarian tumours (Palacios and Gamallo, 1998; Wright *et al*, 1999). Allelic imbalance has not been found in the *β*-catenin gene in ovarian cancers (Nollet *et al*, 1997), but histological grade was not stated in their study. The Tcf-Lef family, members of the HMG-box transcription factors, are expressed in the normal epithelium from different organs, localised to the nucleus or the cytoplasm (Barker *et al*, 1999). To our knowledge, Lef or Tcf has not been described previously in the ovary. We could neither detect nuclear staining in normal OSE nor co-precipitate *β*-catenin/Lef in normal ovarian tissue. We therefore conclude that a complex of *β*-catenin/Lef, for transcriptional regulation, is less likely to be formed in normal OSE.

The function for GSK3*β* and APC in the human ovary is not known. However, as for other epithelial cells, it could be speculated that GSK3*β* and APC regulate the free pool of *β*-catenin in normal ovarian cells. Normal APC function seems to be critical for the colorectal mucosal epithelium, since APC mutations are often found in the earliest stages of colorectal tumorigenesis. The APC protein has been demonstrated in several subcellular compartments including the cytoplasm, nucleus and adhesive cadherin–catenin junctions (Rosin-Arbesfeld *et al*, 2000). In the current study, cytoplasmic and cell border staining of the overlying epithelium (OSE) was present, while nuclear staining was absent. Nuclear APC staining has been described in the uterus using the same antibody as in this study (Nei *et al*, 1999). We believe that APC was not truncated in the normal ovary or in the normal colon sample since the immunoblot demonstrated a band at the expected size of 312 kDa (Smith *et al*, 1993). GSK3 is a serine/threonine specific kinase that was initially shown to phosphorylate and inactivate glycogen synthase (reviewed by Frame and Cohen, 2001). Other targets for phosphorylation have now emerged, that is, *β*-catenin. We have localised GSK3*β* to the cytoplasm and the cell borders of OSE lining the ovary and inclusion cysts. The expression of both GSK3*α* and GSK3*β* were previously described in the normal ovary (Lau *et al*, 1999) at the mRNA and protein level.

In the present study, we found a significant increase of *β*-catenin and GSK3*β* in the adenocarcinomas as compared to normal ovarian tissue and benign adenomas. Adenomatous polyposis coli expression was decreased or absent in ovarian adenocarcinomas, while Lef-1 was constantly expressed in all the tissues analysed. This indicates a possible role for the Wnt-pathway in ovarian tumour cells. Several members of the Tcf-Lef family are expressed in colorectal cancer cells and malignant mammary epithelium (Van de Wetering *et al*, 1996). Nuclear staining for *β*-catenin and Lef-1, indicating dysfunction in normal Wnt-signalling, was found in the OVCAR-3 cells but not in ovarian tumour biopsies. Nuclear *β*-catenin was recently noted in 12 out of 96 serous adenocarcinomas (Lee *et al*, 2003). This could explain why Lef-1, in our study, was found to co-precipitate with *β*-catenin in an ovarian serous adenocarcinoma and in the OVCAR-3 cell line. Constitutive *β*-catenin/Lef-1-mediated transcriptional activity has been detected in four out of 19 investigated ovarian cancer cell lines (Furlong and Morin, 2000).

Beta-catenin has additional functions in the cell since it also binds to the cadherin cell–cell adhesion complex. In fact, both N- and E-cadherin can reduce or block the transcriptional activity of *β*-catenin/Lef1, relocate *β*-catenin to the cell border and inhibit the degradation of *β*-catenin (Sadot *et al*, 1998). We and others have earlier demonstrated that E-cadherin is induced during the progression of ovarian epithelial tumours (Sundfeldt *et al*, 1997; Auersperg *et al*, 2001). The elevated expression of E-cadherin in ovarian cancer could increase binding of *β*-catenin to the plasma membrane. This would then reduce the free pool of cytoplasmic *β*-catenin and inhibit nuclear translocation, reflected by the absence of nuclear *β*-catenin in ovarian tumours in this study. In fact, the absence of E-cadherin can lead to an accumulation of free *β*-catenin and increased transactivation, which can be competed out by transient transfections with E-cadherin (Orsulic *et al*, 1999). Beta-catenin was recently found to be involved in apoptosis (Herren *et al*, 1998). Their finding that caspases have the capacity to cleave *β*-catenin proteolytically at the N- and C-terminal, which will reduce *α*-catenin binding, release actin filaments and reduce cell–cell interaction, indicates that *β*-catenin has an important role at a late stage of apoptosis. Studies on oral squamous cell carcinoma cells also indicate that cadherin-mediated adhesion promotes anchorage-independent growth and suppresses apoptosis (Kantak and Kramer, 1998). A functionally intact cadherin-*β*-*α*-catenin complex in ovarian cancer could make the cancer cells less susceptible to apoptosis and thereby favour growth.

In patients linked to familial adenomatous polyposis, truncations of or mutations in the APC gene are believed to enhance the progression of colorectal or gastric tumours, presumably through stabilisation of *β*-catenin in the cytoplasm (Rubinfeld *et al*, 1996). In the present study, APC expression was demonstrated in normal ovaries and benign tumours, but was low or absent in malignant ovarian tumours. Genetic changes in the APC gene in ovarian tumours have not been described. With the anti-APC NH_2_-terminus antibody, low or absent levels of full-length APC protein were noted in adenocarcinomas and OVCAR-3 cells even though staining of tumour cells was positive. A lower band that might represent a truncated form of APC was demonstrated in these samples. It has been suggested that the NH_2_-terminal fragment of APC is sufficient to localise APC to the apical membranes together with GSK3*β*, Axin, dishevelled and *β*-catenin forming a high molecular weight complex that catalyses the degradation of *β*-catenin (Reinacher-Schick and Gumbiner, 2001). The recently found APC-like gene, APC2, which presented with allelic imbalance in 19 out of 20 ovarian cancers (Jarrett *et al*, 2001), is also noteworthy. It will be very interesting to follow up whether this protein has an impact on Wnt-signalling in the ovary.

Mutational changes of members of the APC/Axin/*β*-catenin complex results in accumulation of cytoplasmic *β*-catenin and nuclear localisation. Nuclear transcriptional activation by *β*-catenin/Tcf-Lef of specific targeted genes, that is, c-Myc and cyclin D, which are upregulated in ovarian tumour, could be important steps in tumour initiation and/or progression (BenZeev and Geiger, 1998). The possible up- or downregulation of suggested targeted genes, however, has not been assessed in this study.

In conclusion, this study demonstrates a significant change in the expression of some components in the Wnt/Wingless-signalling pathway in ovarian epithelial tumours. This could indicate dysfunction of the APC–*β*-catenin–GSK3*β* complex. Lef-1/*β*-catenin precipitates were also found in malignant ovarian cancer, but without concomitant staining in the nucleus of the tumour cells. The proteosomal degradation of *β*-catenin could still be active or *β*-catenin might be focused at the cell–cell junctions via the high E-cadherin expression in ovarian tumours, which in turn could favour tumour growth. In contrast to other tumours, the transcriptional activity of Lef-1/*β*-catenin might play a minor role in ovarian epithelial tumours. Further studies with an *in vitro* approach need to be conducted to elucidate whether the transcriptional activity of *β*-catenin–Lef-1 and/or increased cell-junction stability is of importance in ovarian tumorigenesis.
